# Effects of kinesio tape on kinesiophobia, balance and functional performance of athletes with post anterior cruciate ligament reconstruction: a pilot clinical trial

**DOI:** 10.1186/s13102-020-00203-x

**Published:** 2020-09-14

**Authors:** Milad Gholami, Fahimeh Kamali, Maryam Mirzeai, Alireza Motealleh, MohammadBagher Shamsi

**Affiliations:** 1 Physical Therapy Department, School of Rehabilitation Sciences, Shiraz University of Medical Sciences, Shiraz, Iran; 2 Rehabilitation Sciences Research Center, Shiraz University of Medical Sciences, Shiraz, Iran; 3 School of Allied Medical Sciences, Kermanshah University of Medical Sciences, Kermanshah, Iran

**Keywords:** Anterior cruciate ligament reconstruction, Balance, Kinesio tape, Kinesiophobia

## Abstract

**Background:**

Anterior cruciate ligament trauma is one of the most common knee injuries in professional athletes. This study aimed to investigate the effects of kinesio taping on kinesiophobia, balance, and functional performance in athletes after anterior cruciate ligament reconstruction.

**Methods:**

This randomized, placebo-controlled clinical trial was performed on 20 athletes with anterior cruciate ligament reconstruction (mean age 32.3 ± 6.2 years) at the time of return to sport. The subjects were randomly assigned to the kinesio tape (KT) group (*n* = 10) or placebo KT group (*n* = 10).

While subjects under taped, the following outcomes were measured at baseline, 10 minutes after the intervention, and 2 days later. Kinesiophobia, balance, strength, and functional / agility performance were assessed by the Tampa Scale, Y balance test (YBT), single-leg hops, and 10-yard extremity functional test, respectively.

**Results:**

The results did not show a significant difference between-group post-intervention differences in kinesiophobia (Mean between-group difference = − 6.30, 95% CI = − 4.35 to 1.42, *P*-value = 0.17). Likewise, no significant statistical difference was observed between two study groups in terms of YBT scores (Mean between-group difference ranged over = − 6.30, 95% CI = − 1.1 to 4.7, the effect sizes ranged over = 0.01 to 0.31), *P*-value > 0.05), Single Leg Hop (Mean between-group difference = − 0.48, 95% CI for difference ranged over = − 10.3 to 9.3, effect size = 0.001, *P*-value = 0.918), and 10 Yard test scores (Mean between-group difference = − 0.30, 95% CI = (− 1.3 to 0.75), effect size = 0.02, *P*-value = 0.55) at 2 days after the KT. In the KT and placebo KT groups, RMANOVA indicated that the differences in all variables scores were significant over time with large effect sizes (effect size ranged over = 0.94–0.99; all *P*-value < 0.001).

**Conclusion:**

This study gives no support for any beneficial effect of kinesio taping on the reduction of kinesiophobi or improvement of balance score and functional performance in athletes with post anterior cruciate ligament reconstruction.

**Trial registration:**

This study was registered in the Iranian Clinical Trial Center with the code IRCT20190130042556N1, registered 12 February 2019.

## Background

Anterior cruciate ligament (ACL) trauma is one of the most common knee injuries in professional athletes [1, 2]. The anterior cruciate ligament plays an important role in the stability of the knee joint during running, exercises, and movement of the lower extremity. Furthermore, it prevents forward movement of the tibia in relation to the femur [2]; therefore, trauma which is happened by an injury to the ligament can cause static and dynamic knee instability, reduce the range of motion, decrease balance, and ultimately decrease professional activity [2, 3]. Pain, swelling, and movement limitations, such as the reduction in knee range of motion, strength, and knee function, are other common outcomes after anterior cruciate ligament reconstruction [4].

Kinesiophobia (fear of movement\re-injury) is the most common factor of disability to return to sport, feeling of instability or uncertainty, and ultimately disability to get the pre-injury activity levels after anterior cruciate ligament reconstruction [4]. According to previous case studies, the prevalence of kinesiophobia as a psychological factor that prevents athletes from returning to pre-injury levels after anterior cruciate ligament reconstruction is reported to be between 7 to 30% [5, 6].

Recently, kinesio taping (KT) has been recommended as a non-invasive procedure in the early phase of ACL reconstruction and return to activity. The reason is the convenience of this method as compared to other therapeutic modalities such as TENS, cold therapy, aquatic therapy, and manual therapy. Moreover, this treatment is used as a factor for sports injury prevention, movement pattern improvement, and increased athletes’ performance [2, 7].

Up to now, normalization of muscular function, increasing lymphatic and vascular flow, reduction of pain, contribution to correcting joint malalignments, supporting joints, and improvement of proprioception have been introduced as benefits of KT [8, 9]. There are many theories that justify the effects of KT on muscle activation and joint control, reasoning that KT can stimulate superficial (cutaneous) receptors and modify the motor unit recruitment [7, 10].

As anterior cruciate ligament reconstruction changes sensory and motor components of the knee, KT may also be effective in increasing neuromuscular control of the knee and be a supportive treatment along with other rehabilitation interventions [2, 10]. Despite the widespread use of KT, its mechanism is still unclear and little evidence exists on its effect of this method on post-operative ACL reconstruction in [7, 11–14]. Harput et al. (2016) examined the impact of the knee brace and KT on the performance level of people 6 months after ACL reconstruction. These people felt that they were not able to do activities they used to do prior to cruciate ligament injury due to a fear of movement. In this cross-sectional study, we worked with 30 participants who had an operation 6 months before the tests. The inclusion criteria were to score above 37 on the Tampa Fear Scale. Participants were evaluated in three situations: KT, braces, and no intervention. They were randomly assigned to the groups. Assessments included concentric strength of quadriceps and hamstrings (isokinetic), single-leg hop test, and star excursion balance test. Based on the results, both the KT and the knee brace significantly improved the distance hop and balance level of the subjects. However, only the knee braces could significantly increase quadriceps and hamstring maximum torque. Furthermore, the patients reported better knee performance in a brace and KT than the non-intervention (placebo) group. This study generally demonstrates the positive effect of brace and knee taping on reducing kinesiophobia in people undergoing cruciate ligament reconstruction. It seems that knee brace is more effective than KT in improving knee function [15]. Recently, many studies have published about the effects of kinesio tape (KT) on various problems; such as sports injury, pain reduction, decreased range of motion, and muscle force. However, findings on the effectiveness of KT are conflicting [7, 11, 16]. To the best of our knowledge, no study was found on the effects of knee KT on fear of movement, as a psychological factor in athletes who have done ACL reconstruction (in the phase of return to exercise). Therefore, additional studies are required to evaluate the effectiveness of this method.

The main reason for this study was to consider the effects of knee KT on fear of movement and performance of athletes, who have undergone ACL reconstruction and are in the return to the exercise phase.

## Methods

### Study design and participants

This double-blind randomized controlled trial with parallel groups was conducted in the physiotherapy clinic of Kermanshah Sports Medicine Federation, Kermanshah, Iran.

After obtaining approval from the Ethics Committee of Shiraz University of Medical Sciences with the code of IR.SUMS.REHAB.REC.1397.017 and registration of the trial in the IRCT website under the code of IRCT20190130042556N1, sampling was conducted from January until April 2019.

The inclusion criteria allowed subjects aged 18–45 years, who had a history of ACL reconstruction surgery in the last 6–12 months, were scared to move on the basis of the Tampa questionnaire > 37, were athletes on the basis of the Tegner questionnaire (scores > 5), had full range of motion in hips, knees, and wrists and had a normal gait. They were soccer, futsal, or karate players. Two surgical ACL reconstruction techniques, including hamstring tendon graft and bone-patellar tendon-bone, were performed on them.

Subjects with severe pain, swelling, and range of motion limitation in the knees, dizziness, and involvement of the vestibular system, limiting physical activity (e.g. major orthopedic, neurological or rheumatologic problems in the lower extremities, history of previous surgery in the lower extremity and difference in leg’s length) were excluded. A trained physiotherapist interviewed the eligible participants for confirming the criteria.

**Fig. 1:**
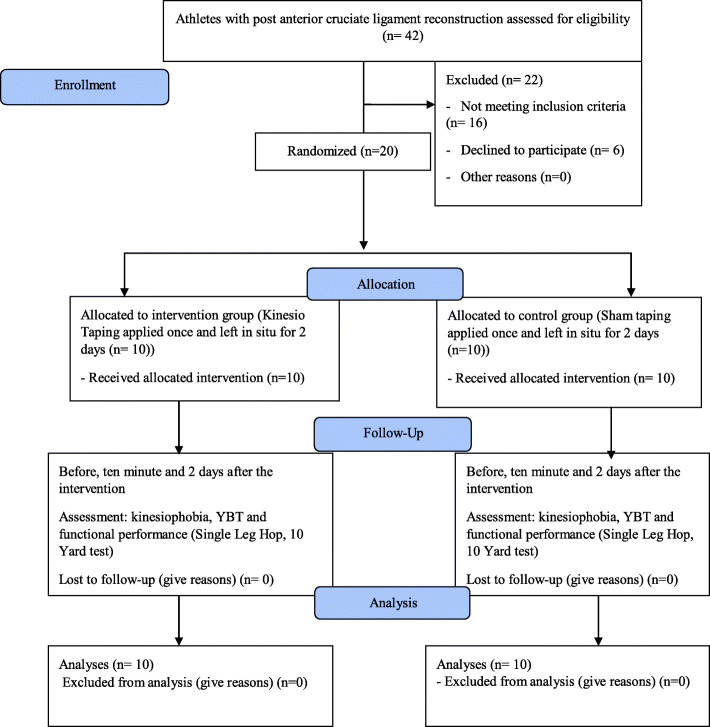
Flow diagram of study selection and data collection process

To estimate the sample size, we did not find any study on our main outcome; therefore, a pilot study was performed at the beginning of our research. The required information on the primary outcome (kinesiophobia) was obtained using a preliminary sample of eight subjects. Considering a difference in the mean (d) = 2, α = 0.05, power of 80%, and Pocock formulae, at least 10 samples per group were computed. Eligible subjects were randomly allocated to KT (*n* = 10) and placebo KT groups (*n* = 10) with a randomized block procedure of block size two using the Random Allocation Software (RAS) version 1.0.0. Random allocation of participants was performed by a statistician not involved in the sampling process. The participants and assessors were blinded to the patients’ allocations (Fig. 1).

Before any intervention, the study goals and the methods were explained for, and written consents were obtained from all participants.

#### Interventions

**Fig. 2:**
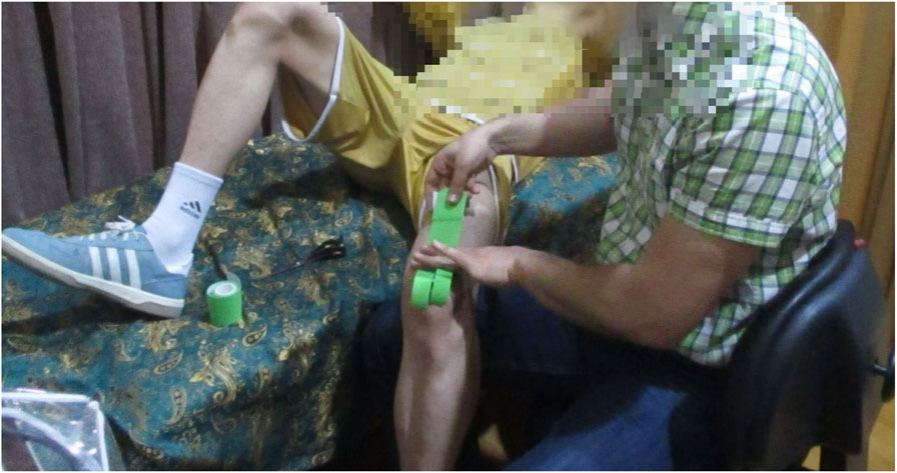
Kinesio tape applications used in the study

Treatment group: To apply the KTs in the intervention group, 5-cm KT length with 50% elongation was attached from the origin to the insertion of the quadriceps muscle, and then it was split to two tails and was come down of two sides of the patella. Then we used an (I) shape tape, which was sized to extend upward from the tuberosity of tibia up to 5-cm above the femoral condyles in order to modify knee movements and stimulate surface receptors. To apply it, by flexing the knee at 80 to 90 degrees, we opened the tape from the middle and put on the tibial tuberosity in full tension. Then the stretch of the tails was reduced to about 50% to attach them to the femoral condyles (Fig. 2).

**Fig. 3:**
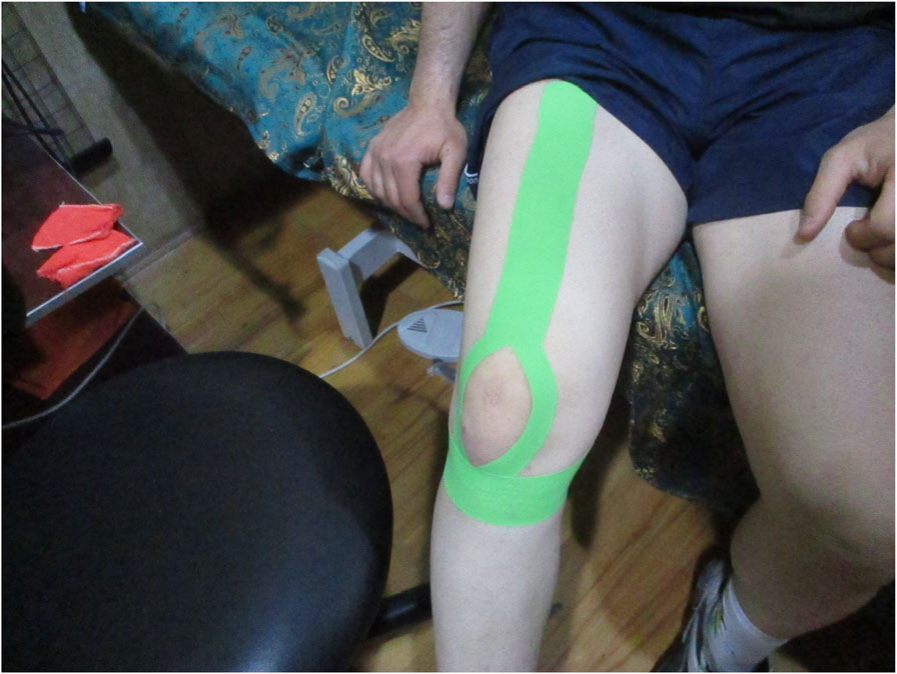
Placebo kinesio tape applications used in the study

Control group: In this group, taping was the same as the treatment group without tension in the tape (Fig. 3). Using KT without tension as a placebo effect may be the most similar intervention to the real KT group (so no need to use a control group without any intervention).

#### Outcome measures

All the outcome measures were evaluated by a blinded assessor, at the baseline, 10 minutes after the intervention, and on the second KT treatment day. At each assessment point, Kinesiophobia (Primary outcome), balance, stability, and functional performance were measured by the Tampa scale, Y balance test (YBT), single-leg hop, and 10-yard extremity functional test (secondary outcomes), respectively.

#### Tampa scale for Kinesiophobia (TSK)

Tampa scale was used to measure the degree of fear of movement (kinesiophobia). This scale was designed by Corey et al. in 1990. It consists of 17 items that every item was scored from 1 (strongly disagree), 2 (disagree), 3 (agree) to 4 (strongly agree). The overall scores are between 17 to 68. The high score shows a greater fear of movement due to pain perception. In this calculation, the score of 37 is the borderline of high and low scores. The Persian version of this questionnaire was developed and validated by Jafari et al. (2010) [17].

#### Y balance test (YBT)

This test which is a modified version of the Star Excursion Balance Test (SEBT) [18] consists of three tapes in three directions for measurement; anterior, posterior-lateral, and posterior-medial. They are stuck to the ground. The posterior tape is positioned 135 degrees from the anterior one, and the two posterior tapes are positioned 90 degrees to each other. For evaluation, the subject was in standing position in the middle of these three lines on one leg without shoes. His foot positioned in the center of the intersection of three tapes and the big toe positioned along the line drawn in the anterior direction. While maintaining a single-leg stance, the person was asked to move his foot forward as far as possible along with these three directions in relation to the stance foot. The distance has been measured by reading the distance to the tip of the foot on the meter. The person repeated the process three times for each direction, and the highest score was recorded during the test. If the subject could not perform the test correctly, the test was repeated until it could be done at least once. If the subject could not do the test properly with six attempts in each direction, it was rejected in that direction. The test results were normalized in all three directions by dividing the distance to the leg length in cm and then multiplied by 100 to obtain the percentile of the length of the lower limb.

$$ YBT=\frac{(anterior)\  or\ \left(\mathrm{posterior}-\mathrm{lateral}\right)\  or\ \left( posterior- medial\right)}{leg\  length}\ast 100 $$

#### Single-leg hop test

This test is one of the valid functional tests for evaluating the knee, which was used to evaluate the objective performance of the athletes. To perform this test, the subject was asked to stand behind a line then position both hands to the back of the body and while maintaining a single-leg stance, try to jump and land on the same foot, as far as he could. The test was repeated three times for each foot and the distance from the tip of the toe in the start line to the base of the toe after landing was measured and recorded with one-millimeter precision. The maximum jump record was taken for each individual subject. Results were also normalized based on each person’s leg‘s length [19].

### Level of functional activity

#### 10-yard lower extremity functional test

**Fig. 4:**
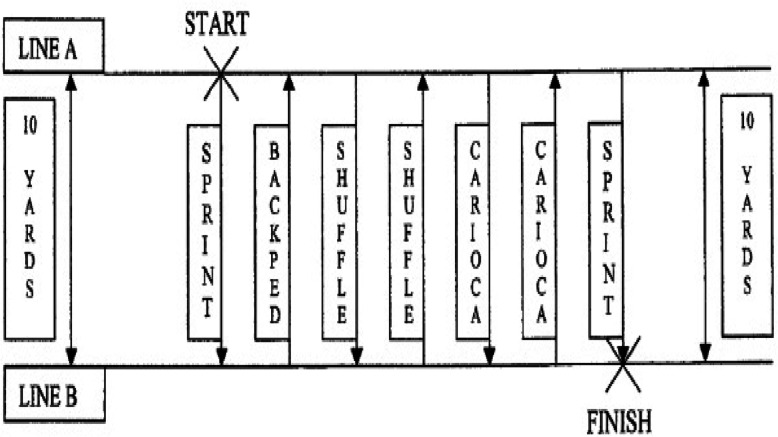
10 Yard Lower Extremity Functional Test

Starting from line A, the subjects sprinted ten yards forward to line B then backpedaled to line A. Next, the subjects side shuffled to line B then side shuffled back to line A. After that subject will carioca to line B and carioca back to line A. Finally, they will sprint through line B. The subjects were asked to make sure to touch each line with their foot (Fig. 4). The administrator measured the time. A normal range of time records for this test for males has been reported to be 17–20 and for females 19–23 s [20].

#### Data analysis

SPSS software version 23.0 (SPSS Inc., Chicago, IL, USA) was used for statistical analyses. Shapiro-Wilk normality test was applied to determine the normality of the distribution of the quantitative data.

Comparisons of baseline characteristics were performed using independent samples chi-squared test and t-test for qualitative and quantitative data, respectively.

The between-group differences for kinesiophobia, functional performance (Single Leg Hop, 10-Yard test, and balance performance) throughout the study were assessed by analysis of Covariance (ANCOVA). Repeated measures ANOVA (RM ANOVA) was used to compare differences in outcome measures before, 10 minutes, and 2 days after the intervention in each group. Mauchly’s test was used to evaluate the assumption of sphericity. All significance was set at *P*-value < 0.05.

### Results

No significant differences were observed for age, height, weight, time of surgery, and Tegner score between the two groups (all *P*-value > 0.05). These data are presented in Table 1.

**Table 1:** Demographic characteristics of the groups

Variables	Kinesiotape group (*n* = 10)	Placebo kinesiotape (*n* = 10)	*p*-value
Age (year)	32.00(5.98)	32.70(6.82)	0.810^**#**^
Height (cm)	177.40(5.13)	179.60(5.87)	0.384^**#**^
Weight (kg)	77.30(11.61)	83(9.83)	0.342^**#**^
BMI (kg/m^2^)	24.47(2.64)	25.41(2.57)	0.772^**#**^
Time of surgery (month)	10.70(1.70)	10.20(2.14)	0.571^**#**^
Tegner Score	7.3(1.49)	6.5(1.58)	0.260^**#**^
Gender (male/female)	9/1	9/1	1^*^

*BMI* Body Mass Index/ Data are means (SD)/ ^**#**^Based on t independent test/ ^*^Based on chi-square test

In the KT group, the changes in all study variables (TSK: F = 34.50; df = 2; *P*-value < 0.001, Single-Leg Hop: F = 50.75; df = 2; *P*-value < 0.001, 10 Yard test (F = 12.61; df = 2; *P*-value < 0.001) and YBT scores were significant over time. Sidak post-hoc test indicated that the differences between the first and the second measurements, as well as between the first and the third measurements were significant (all *P*-value < 0.001).

Likewise, for athletes in the placebo KT group, the results revealed significant changes in the mean value of the TSK (F = 13.41; df = 2; *P*-value < 0.001), Single Leg Hop (F = 21.48; df = 2; *P*-value < 0.001), 10 Yard Test (F = 41.85; df = 2; *P*-value < 0.001) and YBT scores over time (all *P*-value < 0.05), (Table 2). Sidak post-hoc test indicated that the differences between the first and the second measurements (*P*-value < 0.001) as well as between the first and the third measurements (*P* = 0.001) were significant, but the difference between the second and the third measurement was not significant in terms of TSK scores (*P*-value > 0.05).

**Table 2:** Repeated measures ANOVA of mean and standard deviation related to kinesiophobia and functional parameters in three measurements for each group

Variables	Time of measurement period	KT group(***n*** = 10)	PKT group(***n*** = 10)
		Mean (SD)	Mean (SD)
TSK	Before KT	42.40 (6.65)	44.40(6.20)
	10 min intervention	37.90(3.87)	41.80(5.81)
	2th KT day	34.90(4.81)	39.30(2.91)
	* Repeated measures ANOVA (inter-group)	F = 34.50; df = 2; *P*-value < 0.001	F = 13.41; df = 2; *P*-value < 0.001
Single Leg Hop	Before KT	176.69(35.12)	175.67(31.09)
	10 min after intervention	190.133(34.00)	189.44(24.16)
	2th KT day	197.01(44.01)	196.29(22.71)
	* Repeatd measures ANOVA (inter-group)	F = 50.75; df = 2; *P*-value < 0.001	F = 21.48; df = 2; *P*-value < 0.001
10 Yard Test	Before KT	26.07(4.53)	24.94(1.35)
	10 min after intervention	24.67(3.63)	23.47(1.34)
	2th KT day	23.31(2.81)	22.97(1.27)
	* Repeated measures ANOVA (inter-group)	F = 12.61; df = 2; *P*-value < 0.001	F = 41.85; df = 2; *P*-value < 0.001
YBT (Anterior reach)	Before KT	100.04(10.84)	96.95(5.47)
	10 min after intervention	105.01(8.82)	100.22(5.63)
	2th KT day	105.55(8.63)	105.45(6.01)
	* Repeated measures ANOVA (inter-group)	F = 14.92; df = 2; *P*-value < 0.001	F = 27.21; df = 2; *P*-value < 0.001
YBT (Posteromedial reach)	Before KT	97.51(13.08)	98.57(9.38)
	10 min after intervention	102.30(11.44)	104.85(11.23)
	2th KT day	107.37(9.24)	107.41(9.52)
	* Repeated measures ANOVA (inter-group)	F = 17.79; df = 2; *P*-value < 0.001	F = 43.31; df = 2; *P*-value < 0.001
YBT (Postero-lateral reach)	Before KT	92.63(9.59)	96.04(5.54)
	10 min after intervention	99.98(8.77)	100.05(5.13)
	2th KT day	105.05(8.72)	103.39(6.97)
	* Repeated measures ANOVA (inter-group)	F = 67.75; df = 2; *P*-value < 0.001	F = 24.28; df = 2; *P*-value < 0.001

*KT* kinesio tape, *PKT* placebo kinesio tape, *TSK* Tampa Scale for Kinesiophobia, *YBT* Y Balance Test; Mean (SD) was reported;*P value from Greenhouse-Geisser test has been reported based on the results of Mauchly’s test

In the other words, the KT group and placebo KT group reduced TSK scores with a large effect size that reached statistical significance over time (effect size ranged over = 0.97–99; *P*-value < 0.001). In both groups, improvement in YBT scores (effect size ranged over = 0.98–99; *P*-value < 0.001), 10 Yard Test (effect size ranged over = 0.97–99; *P*-value < 0.001), and Single Leg Hop (effect size ranged over = 0.94–98; *P*-value < 0.001) reached statistical significance over time.

Results revealed no significant difference between-group post-intervention in any of the studied variables. In the other words, based on results of ANCOVA for examining the effects of KT (compared to the placebo KT) on the variables, the change in TSK score from the first to the second measurements was not significantly different (Mean between-group difference = − 3.55, 95% CI for difference = − 5.01 to 2.09, effect size = 0.35). The changes of the scores from the first to the third measurements of TSK score were not significantly different in the KT group as compared to the placebo KT group (Mean between-group difference = − 6.30, 95% CI for difference = − 4.35 to 1.42, effect size = 0.42) (Table 3).

**Table 3:** Comparison of changes in kinesiophobia and functional parameters among the 2 groups of study

Variables	Difference between stages of measurement	Mean Between-Group Difference	95% CI for Difference	^**#**^*P* value (between groups)	Effect size (between groups)
TSK	Second measurement to first measurement	−3.55	(−5.01 to 2.09)	0.254	0.354
	Third measurement to first measurement	−6.30	(−4.35 to 1.42)	0.172	0.419
Single Leg Hop	Second measurement to first measurement	0.95	(−8.66 to 6.76)	0.798	0.004
	Third measurement to first measurement	−0.48	(−10.32 to 9.34)	0.918	0.001
10 Yard Test	Second measurement to first measurement	0.298	(−0.37 to 0.96)	0.360	0.05
	Third measurement to first measurement	−0.30	(−1.35 to 0.75)	0.550	0.021
YBT (Anterior reach)	Second measurement to first measurement	2.40	(−0.82 to 5.62)	0.134	0.127
	Third measurement to first measurement	−1.10	(−5.28 to 3.08)	0.585	0.018
YBT (Posteromedial reach)	Second measurement to first measurement	−1.54	(−4.66 to 1.58)	0.313	0.060
	Third measurement to first measurement	0.76	(−3.14 to 4.66)	0.687	0.010
YBT (Postero-lateral reach)	Second measurement to first measurement	2.81	(0.05 to 5.58)	0.434	0.214
	Third measurement to first measurement	4.72	(1.12 to 8.31)	0.986	0.311

*TSK* Tampa Scale for Kinesiophobia, *YBT* Y Balance; Mean between-group difference and 95% CI for difference were reported/ ^#^*P* value is reported based on the analysis of covariance

Regarding YBT after intervention measurement scores, no significant differences between groups were observed (Mean between-group difference ranged over = − 6.30, 95% CI for difference = − 1.10 to 4.7, effect size ranged over = 0.01 to 0.31), *P*-value > 0.05). Likewise, the two study groups had no significant statistical differences in terms of Single Leg Hop (Mean between-group difference = − 0.48, 95% CI for difference ranged over = − 10.32 to 9.34, effect size = 0.001, *P*-value = 0.918), and 10 Yard test scores (Mean between-group difference = − 0.30, 95% CI for difference = (− 1.35 to 0.75), effect size = 0.021, *P*-value = 0.550) at 2 days after the KT (all *P*-value > 0.05) (Table 3).

## Discussion

The aim of this study was to examine the effects of KT (compared to placebo KT) on kinesiophobia, balance, and functional performance of athletes with post anterior cruciate ligament reconstruction. The results did not show any significant difference between-group post-intervention in any of the study variables. In the other words, between groups comparisons identified a small effect size for all study variables (effect size ranged over = 0.01 to 0.42) that did not reach significance at the 0.05 level (all *P*-value > 0.05). The small to medium effect size (effect size < 0.5) and lack of statistical significance in the comparison between the KT and the placebo KT groups, are most likely due to a lack of sufficient sample size and statistical power. Though changes experienced by the KT group in the present pilot trial may be clinically useful, in the case of this particular outcome measures, the power obtained was not sufficient to produce significant results. Furthermore, the lack of statistical significance in the comparison between groups, maybe indicate that a 2-day KT period is not sufficient timeframe to demonstrate significant effects; so there is a need to further explore the clinical significance of these data.

The results of the present study showed that after KT, TSK score, athletes’ performance, and balance were improved significantly in both groups overtime with large effect size. It seems that the improvement in outcome measures is related to the psychological effect of KT in both groups.

For the variable of fear of movement, re-injury in the KT group and the placebo KT group, the score of the Tampa Fear Scale decreased significantly compared to the pre-intervention level, but the level of improvement and reduction of fear of movement in the KT group was more than the placebo KT group.

These results showed that after ACL reconstruction, applying KT on the knees of the athletes, reduced the fear of re-injury significantly.

Patients who suffer from musculoskeletal injury are at risk of biopsychosocial impairments, and kinesiophobia has been reported as one of a variety of biopsychosocial types [21, 22]. In patients with cruciate ligament injury kinesiophobia is more likely to occur in active individuals whose physical damage has improved physiologically but still prevents them from returning to competitive exercise or higher levels of performance [21, 22]. It has been suggested that reducing pain-related fear can lead to pain reduction because the fear of pain is more debilitating than the pain itself [23, 24].

Previous studies have considered the effect of kineso taping on kinesiophobia in patients with musculoskeletal pain. It seems that using KTs, by instilling a sense of confidence and support in the joint, makes the person ready psychologically to return to the exercise and reduces their fear of movement and injury. We suspect that the small difference between the two groups and the fact that they both benefited from these positive effects is due to the psychological effect of KT in both groups (treatment group with proper stretching and placebo one without stretching). This reduced fear and increased self-esteem can also be a reason for improving other individual variables such as balance, agility, and performance level.

In the Hoffman et al. study, which examined the effects of KT on reducing kinesiophobia in patients with musculoskeletal problems, there was enough evidence to suggest that kinesiophobia was reduced by the use of KT in comparison with placebo KT, which is inconsistent with our results [25]. This difference may be due to the different KT techniques, which were used in the placebo group in these studies.

In both groups, after KT, athletes’ performance and balance were improved significantly, but the results did not show a significant difference between these groups. The results of 10-yards lower extremity test as a criterion for evaluating athletes’ agility showed that in both treatment and control groups, the test scores decreased significantly during the intervention. Since the 10-yard lower limb test score is the time taken to perform the test in seconds, its reduction is a sign of improvement of agility in athletes, and it shows that KT can play an important role in increasing agility in athletes following anterior cruciate ligament reconstruction.

In this study, in order to evaluate the subjects’ functional status, the single-leg hop test was used. According to the results, the test’s scores in the evaluations of the immediate stage (10 minutes after the baseline) and short-term stages (2 days after the baseline) were increased in both groups. In this study, increasing the score of the single-leg hop test, which is jumping distance in meters, proves that the practice of KT can significantly improve the performance of the athletes after anterior cruciate ligament reconstruction. The main reasons for the improvement in the single-leg hop test are reducing pain and increasing joint proprioception, which are followed by the use of KT.

Oliveira et al. (2014), executed research on patients with ACL rupture after reconstruction in which neuromuscular functions of the quadriceps and balance were evaluated. They reported that there were not any changes in the performance or balance of the KT group before and after the intervention as well as the placebo group, which is inconsistent with our results. Again, it is possible that the KT technique and their stretching rate were different in these studies [13].

Moreover, Herrington (2004), studied the effect of patellar taping on the single-leg hop test in healthy people, and he reported that no significant effect was observed [26]. The reason for the results of Herrington’s study can be attributed to the health status of the subjects. Therefore, there may be no pain that the KT reduces and improve the single-leg hop test [26].

In our study, the comparison between different time stages (immediate and short-term evaluations) showed a significant improvement in both treatment and control groups in all three directions of balance. Generally, in both groups and in all directions, the trend of change in the balance of the athletes at different times was similar and increasing.

These results were inconsistent with the results of Oliviera’s study, which has reported that KT did not change the balance level of those people who were undergoing anterior cruciate ligament reconstruction. The reason for the difference in the results of these two studies could be attributed to the way of evaluation of the level of balance in participants. Unlike the present study, which used a modified star excursion test, Oliviera et al., chose a baropodometric method to assess their patients’ balance. Also, the evaluations in that study were done just after KT and if longer-term evaluations were made, it may be there were other results. In addition, in the current study, the KT was applied to the knee joint which could provide more mechanical support and stability than the method of KT used in Oliviera’s study that only the quadriceps muscle was taped. This mechanical stability can increase the balance in the subject [13].

The results of the present study regarding the balance variable are also in line with the results of the study by Harput et al., who reported a significant effect of KT on balance level and star excursion test score after anterior cruciate ligament reconstruction [15].

In spite of the contradictory results in various studies, still, the main finding is the positive effect of KTs on improving physical abilities.

### Limitations

Main limitations of the current pilot study are the relatively small sample size and short follow-up period due to the impossibility of long-term continuation of the study and the limited time for evaluations; thus, the sample size and follow-up period of the current trial might not have been sufficient to be able to find significant differences between the groups in terms of the change in study variables (TSK, Single-Leg Hop, 10 Yard Test, and YBT scores). However, it had some strengths such as its double-blind, randomized, and placebo-controlled design. Also, for further RCTs, this pilot study may be provided preliminary data for power analysis and sample size calculations.

## Conclusion

This study gives no support for any beneficial effect of kinesio taping on the reduction of kinesiophobi or improvement of balance score and functional performance in athletes with post anterior cruciate ligament reconstruction. The changes in all study variables between the first and the last measurements in both groups (regarding that the control group was a placebo KT one) and lack of the difference between the two groups may be due to the psychological effects of using the KT. To discover the effect of kinesio taping on kinesiophobi and the clinical significance of these data, further, well-designed and robust clinical trials with adequate power and longer-term follow-up in combination with other therapeutic and rehabilitation interventions are suggested.

## Data Availability

The datasets used and/or analyses during the current study are available from the corresponding author on reasonable request.

## Abbreviations

ACL: Anterior cruciate ligament
RMANOVA: Repeated measures ANOVA
SEBT: Star Excursion Balance Test
TSK: Tampa Scale for Kinesiophobia
YBT: Y Balance Test
